# Nucleic Acid Immunity in the Pathogenesis of Cutaneous Lupus Erythematosus

**DOI:** 10.3389/fimmu.2019.01636

**Published:** 2019-07-16

**Authors:** Claudia Günther

**Affiliations:** Department of Dermatology, University Hospital Carl Gustav Carus, Technical University Dresden, Dresden, Germany

**Keywords:** nucleic acid immunity, cutaneous lupus erythematosus, innate immunity, nucleic acid sensors, type I interferon

## Abstract

Cutaneous lupus erythematosus can be a devastating painful and mutilating disease that is associated with an inflammatory response in the skin driven by type I interferon activation. Clearance defects in the extra- and intracellular space lead to an enhanced prevalence of nucleic acids that represent danger signals for the innate immune system. Self nucleic acids can stimulate DNA and RNA sensors that have originally evolved to ensure viral defense. Their activation can induce a type I interferon dominated response in resident skin cells, macrophages and dendritic cells that subsequently progresses to adaptive immune stimulation. The genetic exploration of rare monogenic type I interferon driven diseases helped to identify these pathogenic concepts. Based on a genetic susceptibility lupus patients are more vulnerable to environmental trigger factors such as UV-irradiation that can provoke inflammation with local tissue destruction and eventually systemic disease. Understanding of these pathogenic concepts is a prerequisite for development of targeted therapies.

## Introduction

Lupus erythematosus is a chronic relapsing autoimmune disease with a varying spectrum of clinical manifestations ranging from sole cutaneous involvement to fatal multi-organ disease (1). The wide clinical range of cutaneous symptoms includes a diverse spectrum from acute rash, discoid lupus, cold induced chilblain lesions to succulent subcutaneous infiltrates resembling lupus tumidus (2, 3). Cutaneous lupus can occur with or without systemic involvement. Patients with systemic lupus erythematosus (SLE) frequently develop skin lesions. Disease flares can be induced by external trigger factors among which viral infections, UV-irradiation and smoking are the most important (1). Cutaneous inflammation is characterized by a perivascular and periadnexial lymphohistiocytic infiltration accompanied by an interphase dermatitis and variable epidermal involvement. This lymphocyte activation is a hallmark of adaptive immune stimulation. T cells provide help for activation of autoantigen specific B cells and producing autoantibodies. In patients with lupus erythematosus the target structures of these autoantibodies are proteins associated with nucleic acids (1). Immune complexes containing autoantibodies to nucleic acid binding proteins and nucleic acids are frequently deposited along the basement membrane zone of the skin and the concentration of autoantibodies against double stranded DNA (dsDNA) correlates with systemic involvement and disease activity. This clinical association points to the importance of the immune reaction to nucleic acids during the course of disease.

Cutaneous lupus lesions are characterized by prominent upregulation of type I interferons (IFN) and type I IFN induced proteins and chemokines in the skin (4). Type I IFNs are primarily produced during innate immune recognition of viral nucleic acids for antiviral defense. They lead to a broad immune stimulation know as antiviral state that can overcome immune tolerance mechanism.

This review aims to provide an overview on the role of innate immune processes caused by disturbances in the normal restriction and regulation of the nucleic acid metabolism in the pathogenesis of cutaneous lupus erythematosus.

## Nucleic Acid Sensing

**Figure 1:**
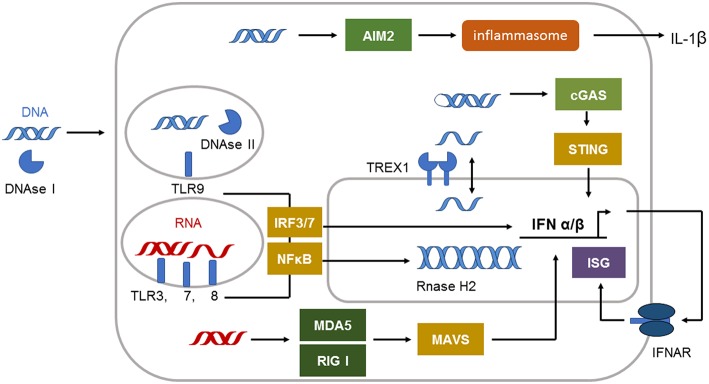
Immune sensing pathways for RNA and DNA in the cell. The compartmentalization of nucleic acids in the cells is strictly regulated. Unrestricted RNA and DNA in the cytosol is a danger signal and can be sensed by specific receptors. The cytosolic immune receptor cyclic GMP–AMP synthetase (cGAS) detects long double-stranded DNA (dsDNA) or highly structured single-stranded DNA (ssDNA) in a special conformation. cGAS signals via STING to induce type I IFN induction. dsDNA can also be recognized by the inflammasome sensor AIM2 leading to IL-1β secretion. Cytosolic DNA is efficiently degraded by 3′ repair exonuclease 1 (TREX1) located in the outer nuclear membrane. DNAse II is restricting DNA in the endolysosome. Endosomal DNA can be detected by Toll-like receptor 9 (TLR9). TLR9 signals via interferon (IFN)-regulatory factor 7 (IRF7) and nuclear factor-κB (NF-κB) to induce type I IFN. dsRNA is detected by TLR3 and TLR7, whereas single-stranded RNA (ssRNA) is sensed by TLR7 and TLR8 in the endosome. These receptors signal via adaptor proteins to induce interferon (IFN)-regulatory factor 3 (IRF3)–IRF7 and type I IFN production and via nuclear factor-κB (NF-κB) to induce proinflammatory cytokines. The cytosolic sensors melanoma differentiation associated gene 5 (MDA5) and retinoic acid inducible gene I (RIG-I) detect dsRNA. Both signal through mitochondrial antiviral signaling protein (MAVS) to induce type I IFN production.

The defense against viral infections is in great part dependent on recognition of viral nucleic acids by pattern recognition receptors (PRRs) of the innate immune system (5). They developed during millions of years to protect primates and their ancestors from viral attack. The system of PRRs includes membrane, endoplasmic, and cytoplasmatic subtypes specific for either ribonucleic acid (RNA) or deoxyribonucleic acid (DNA) (6) (Figure 1). Cell surface and endosomal membranes of most cells are equipped with Toll like receptors (TLRs) that recognize bacterial wall components or nucleic acids. TLR3 is located at the cell surface of some tissue cells such as fibroblasts and in the endosome to recognize dsRNA. The endosomal receptors TLR7 and TLR8 recognize single stranded RNA. TLR9, expressed by plasmacytoid dendritic cells and B cells, is the only TLR recognizing dsDNA in the endosome. In the cytosol the RIG-I-like receptor (RLR) family of RNA sensors (retinoic acid inducible gene I (RIG-I) and melanoma differentiation associated gene 5 (MDA5 or IFIH1) recognize short or long stretches of dsRNA, respectively (6).

The molecules absent in melanoma 2 (AIM2), cyclic GMP–AMP synthetase (cGAS) or IFN inducible protein 16 (IFI16) can sense dsDNA in the cytosol (Figure 1). cGAS binds to dsDNA but can also be activated by structured ssDNA (7, 8). cGAS activation catalyzes the synthesis of cyclic GMP-AMP (cGAMP) from ATP and GTP (9). The second messenger cGAMP binds to the endoplasmic-reticulum (ER)-membrane adaptor stimulator of interferon induced genes (STING) and induces trafficking of STING to the ER/Golgi apparatus where it activates the tyrosine binding kinase 1 (TBK1) (9) (Figure 1). TBK1 phosphorylates the transcription factor IRF3 that enters the nucleus and induces type I IFN expression (9).

Activation of AIM2 by long dsDNA leads to oligomerization of AIM2 and subsequent formation of an inflammasome in the cytosol. The activation of the AIM2 inflammasome results in caspase 1 activation and the release of the proinflammatory cytokine IL-1beta (10, 11) (Figure 1). The dsDNA sensor IFI16 is located in the nucleus under steady state conditions but can be translated to the cytoplasm following UV-irradiation (12). IFI16 mediated recognition of viral DNA can also induce inflammasome formation and IL-1beta activation (12). In addition, during herpes simplex virus type 1 infection IFI16 was described to interact with STING leading to IRF3 dependent type I IFN induction (12). However, other reports describe that IFI16 is dispensable for type I IFN induction following cytomegalovirus infection (13).

Type I IFN is the dominating cytokine induced in the signaling cascade of most nucleic acid sensing PRRs. They directly or indirectly induce transcription factors that upregulate the expression of antiviral effector proteins, chemokines and cytokines mediating the antiviral defense (6). Type I IFNs include multiple subtypes such as IFNbeta and IFNalpha subtypes, depending on species, as well as the lesser-known IFNkappa secreted by keratinocytes (14). Type I IFNs can be produced by all nucleated cells and all cells can respond to type I IFNs through the type I IFN receptor (IFNAR), which binds all subtypes (15) and translates its signal through the Janus kinase 1 and tyrosine kinase (Tyk) 2 (16).

All of these antiviral sensors are not specific for viral nucleic acids but can also be activated by self RNA or DNA (14). This is relevant for an induction of anti-tumor immunity. Self-DNA from dying tumor cells has been shown to induce a STING-dependent immunity that is important for induction of a type I IFN stimulated anti-tumor defense (17, 18).

Under steady state conditions the DNA metabolism needs to be tightly regulated to prevent immune activation by self nucleic acids that would result in autoimmunity (6). Several nucleases and nucleic acid modifying enzymes ensure this task. Normally, DNA is located in the nucleus and mitochondria. DNAse I degrades free DNA that reaches the extracellular space. DNAse II eliminates intracellular DNA from the lysosomal compartment and DNAse III (also designated TREX1) protects the cytoplasm from DNA accumulation. In the nucleus, free ssDNA is bound by RAD51, and replication protein A (RPA) to prevent its translocation to the cytoplasm (19).

Any disturbance in the tightly regulated nucleic acid metabolism and the antiviral defense can lead to chronic type I IFN stimulation that will continuously alert the immune system resulting in an antiviral state. In genetically susceptibly individuals such chronic immune stimulation can lead to the development of autoimmunity.

## Lessons Learned From Monogenic Diseases

Monogenic diseases are caused by a single mutation in a respective gene. This allows to understand the role of a mutated protein in the disease process and its impact on the clinical phenotype. The first reported monogenic diseases that helped to elucidate the pathogenesis of SLE were complement deficiencies (20). Homozygous deficiency in C1q is associated with childhood onset of classical discoid lupus, Raynaud's phenomenon, non-scarring alopecia, oral ulceration, and cerebral vasculitis (21, 22). A loss of complement supports the formation of large insoluble immune complexes formed by nucleic acids and autoantibodies (20). These immune complexes facilitate the uptake and recognition of nucleic acids by immune cells (23).

In the last years, monogenic diseases associated with type I IFN upregulation were described as interferonopathies (24). Interferonopathies are caused by mutations that often affect genes involved in nucleic acid metabolism and nucleic acid sensing pathways. Only some mutations have been clinically associated with lupus. The other cause specific disease phenotypes that can share clinical signs and symptoms such as cutaneous ulcerations or livedo with lupus (25). Understanding the pathogenesis of such interferonopathies might help to elucidate the multiple pathogenic pathways that can cause the wide clinical spectrum of lupus erythematosus (26, 27).

### Aicardi Goutières Syndrome

One of the main interferonopathy leading to an improved understanding of the induction of autoimmune disorders and especially lupus is Aicardi Goutières syndrome (28, 29). Aicardi Goutières syndrome is a rare encephalopathy of childhood and infancy that may mimic congenital viral infection (29). Affected children suffer from early onset of seizures and fever leading to mental retardation. Many patients show features of systemic autoimmunity especially SLE such as antinuclear and anti-DNA autoantibodies, arthritis as well as cold induced chilblain lupus lesions on acral locations (30).

Aicardi Goutières syndrome is genetically heterogeneous and can be based on mainly biallelic mutations in 7 different genes, that are all involved in nucleic acid metabolism or enhanced type I interferon sensing (31). The first mutations described were detected in the three prime repair exonuclease 1 (Trex1, DNase III) (32). TREX1 is a cytoplasmic exonuclease that degrades ssDNA as single oligonucleotides or overhangs in dsDNA (33, 34). It safeguards the cytosol from accumulation of nucleic acids and thereby prevents the activation of innate nucleic acid sensors by self DNA (33). TREX1 deficiency causes cellular stress and DNA damage response (33). During this process, ssDNA that cannot be retained in the nucleus due to exhaustion of the nucleic binding capacity, escapes into the cytosol and stimulates a cGAS dependent chronic type I IFN response (19, 35). Heterozygous TREX1 mutations enhance the risk for the development of SLE (36).

Other mutations inducing Aicardi Goutières syndrome affect any of the three subunits of the ribonuclease H2 (RNaseH2) (37). The nuclear enzyme is responsible for removing ribonucleotides misincorporated in the DNA and acts on RNA/DNA hybrids (38). Mutations in RNaseH2 lead to cell stress and an increase in DNA damage that is associated with an increased type I interferon production predisposing to autoimmunity and SLE (39).

Further mutations can affect the SAM Domain and HD Domain 1 (SAMHD1) controlling the building blocks of DNA, the nucleotide triphosphate pool in the cell (40). Mutations can also affect the adenosin deaminase, RNA-specific (ADAR), important for RNA editing and gain of function mutations of the cytosolic RNA sensor MDA5 (IFIH1) (31). Mutations in ADAR have been described associated with discoid lupus erythematosus (41) and IFIH1 is a risk gene for SLE (42).

### STING-Associated Vasculopathy, Infantile-Onset

STING-associated vasculopathy, infantile-onset (SAVI) is an autoinflammatory disease starting in early childhood with a vasculopathy causing erythematous, pustular, or blistering infiltrates with scaling on acral locations such as the cheeks, ears, nose, and digits that may even lead to necrotizing skin lesions resulting in mutilation and scarring (43). The symptoms worsened in cold weather. Patients may develop low-grade fever flares and inflammatory interstitial lung disease associated with lung fibrosis. They also had low titer antinuclear or antiphospholipid autoantibodies. The disease is caused by a heterozygous *de novo* gain of function mutation in STING-signaling resulting in a constitutive activation of the IFNB promoter and upregulation of type I IFN stimulated genes (ISG) in blood. Interestingly, a single family with SAVI and lupus-like features due to a dominant STING mutation was reported (44).

### Familial Chilblain Lupus

Familial chilblain lupus is a rare autosomal dominant monogenic form of lupus erythematosus based on heterozygous mutations in TREX1, SAMHD1, or STING (45–47). The disease is characterized by cold induced livoid infiltrates on acral locations that tend to ulcerate and occur since early childhood (48, 49). Systemic involvement is possible and includes arthritis, antinuclear antibodies, and cytopenias. Type I IFNs are upregulated in skin and blood of the patients (50, 51). Patient fibroblasts with TREX1 mutation respond to cold exposure with cellular stress, senescence, and ISG induction (52).

### CANDLE Syndrome

Chronic atypical neutrophilic dermatosis with lipodystrophy and elevated temperature (CANDLE) is an autosomal recessive inherited autoinflammatory disease (53). Starting in infancy, patients suffer from fever accompanied by a widespread, violaceous and often annular, cutaneous eruption. Further symptoms are partial lipodystrophy, hepatomegaly, joint contractions with muscle atrophy and arthralgias. An interferon signature has been found in blood of affected patients. CANDLE is caused by homozygous mutations in PSMB8 encoding proteasome subunit β type 8, which functions as the chymotrypsin-like catalytic subunit of the immunoproteasome and is involved in processing of antigens presented by MHC class-I (54). A recent genome wide imputation approach identified a rare variation in PSMB8 as candidate gene for SLE in a European ancestry population (55).

### Childhood-Onset Polyarteritis Nodosa—ADA2 Deficiency

Childhood-onset polyarteritis nodosa is an autosomal recessive disease caused by biallelic mutations of CECR1 (cat eye syndrome chromosome region, candidate 1), encoding extracellular deaminase 2 (ADA2) that deaminates adenosine to inosine (56). A defect in this nucleosid modification induced monocyte-macrophage polarization toward the M1 subset. This inflammatory subset impaired vascular integrity (56). All the patients presented with recurrent fevers and livedo racemosa in early childhood (56, 57). Cutaneous involvement included erythematous nodules and plaques that healed with scars or tissue necrosis leading to mutilation.

### DNAse I

Homozygous mutations in DNAse I impair restriction of DNA in the extracellular space. Affected patients suffered from childhood onset SLE with fever, generalized rash, kidney involvement, and prominent autoantibodys directed against dsDNA, and Ro (58). Furthermore, mutations in one of the three human homologs of DNase I, DNase 1L3, caused SLE with onset during childhood, autoantibody formation and nephritis (59).

### DNAse II

Patients with biallelic mutations in DNase II and a functional loss of this lysosomal endonuclease develop an autoinflammatory disease state with pancytopenia, deforming arthropathy, glomerulonephritis, mild learning difficulties in school, cutaneous ulcerations, and vasculitis like skin lesions starting in early childhood (60). Deficiency of DNase II in fibroblasts was associated with enhanced expression of ISGs. This ISG upregulation was also detectable in blood indicating the importance of type I IFN in the induction of the clinical symptoms. Variants in DNaseII showed weak associations with the risk of nephritis among korean SLE patients (61).

## Implications for Multifactorial Cutaneous Lupus Erythematosus

Healthy skin has a regular turnover of keratinocytes and is continuously exposed to cell damaging influences such as irradiation. The daily occurring ubiquitous cell debris needs to be safely eliminated by normal immune processes. This process is ensured by several mechanisms that are crucial for the maintenance of self tolerance (1). Phagocytes can recognize and engulf apoptotic cells by altered cell membrane components or self antigens opsonized by autoantibodies or complement components (1). In addition, nucleases degrade circulating nucleic acids. Defective clearance mechanisms result in secondary necrosis and release of self antigens that can be accessed by innate immune cells (62). Lesional lupus skin biopsies characteristically show an interface dermatits with a varying number of dyskeratotic dying keratinocytes. It has been described that such apoptotic cell debris was present for prolonged time in lupus lesions indicating a defect in apoptotic clearance (63). Due to a defect in phagocytes of SLE patients such apoptotic debris can be found in germinal centers attached to follicular dendritic cells that can present autoantigens (64, 65). In addition, mutations in the genes encoding the complement components and impairing regular clearance are among the strongest risk factors for the development of SLE including cutaneous involvement (20). Furthermore, defects in DNAse I, that breaks down extracellular DNA, are associated with lupus erythematosus (66).

**Figure 2:**
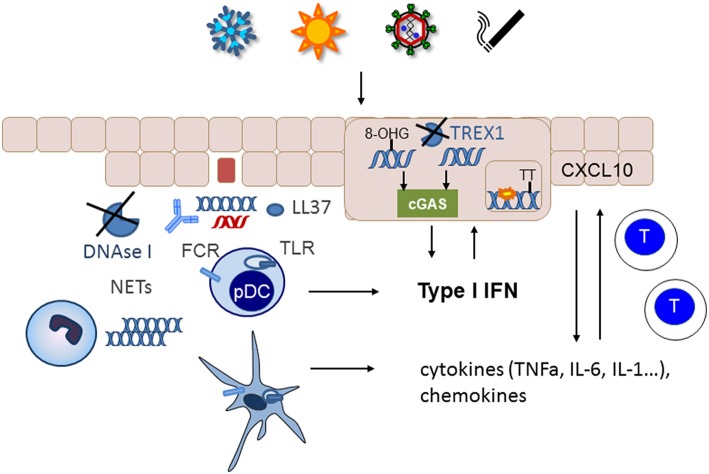
Nucelic acid immunity in the pathogenesis of cutaneous lupus erythematosus. Cutaneous lupus lesions frequently show apoptotic keratinocytes and nucleic acids complexed with autoantibodies along the basement membrane zone (blue DNA, red RNA). In patients with defects in nucleic acids clearance such as complement defects or impaired function of DNase I these nucleic acids can be taken up by immune cells such as plasmacytoid (pDC) or myeloid dendritic cells and sensed by TLRs. Neutrophils can extrude additional DNA into the extracellular space. Phagocytosis of nucleic acids is enhanced by immuncomplexes, complexation with antimicrobial peptides such as LL37 and supported by FC-receptors. The resulting cytokine and chemokine production stimulates an adaptive immune response with infiltration of T cells into the skin. Environmental trigger factors such as UV-irradition or smoking induce DNA damage and reactive oxygen species that can cause oxidation of DNA. The 8-hydroxyguanosine (8-OHG) modification protects DNA against degradation by TREX1. The unrestricted DNA can accumulate in the cytoplasm and induce cGAS stimulation leading to type I IFN induction. In patients with deficiency in TREX1 intracellular DNA can accumulate as danger signal. Other mutations such as RNAseH2 cause ribonucleotide accumulation in DNA that predisposes to cyclobutanpyrimide formation after UV-irradiation and thereby enhance cellular stress.

If nucleic acids are not removed from the extracellular space, they can serve as autoantigens. Especially if they are complexed with antimicrobial peptides such as LL37, autoantibodies, or the architectural chromosomal protein and proinflammatory mediator high mobility group box protein 1 (HMGB1), nucleic acids are immunogenic, protected from degradation and foster the uptake by monocytes/macrophages and dendritic cells (67–70) (Figure 2). The latter engulf immune complexes in their endosomal compartments where they can access nucleic acid sensing TLRs (Figure 2). Monocytes and myeloid dendritic cells can sense RNA by TLR 7 and 8 that results in NFkappa B and TNF alpha upregulation (71). Recognition of HMGB1 nucleosome complexes by TLR2 can further potentiate this cytokine induction that mediates the stimulation of the adaptive immune system (70). TLR3, expressed by tissue resident keratinocytes and fibroblasts senses self RNA that can lead to local type I IFN induction. Plasmacytoid dendritic cells can sense dsDNA by TLR9 and stimulate a robust type I IFN response after the uptake of immune complexes or DNA bound to antimicrobial peptides (23). An accumulation of plasmacytoid dendritic cells was reported in certain types of cutaneous lupus (72) (Figure 2).

In addition to unrestricted cellular debris as source of extracellular self-nucleic acids an intense active extrusion of DNA by leukocytes has been described in SLE patients (73). These neutrophil extracellular traps are a known danger signals in SLE and their deposition has also been described in cutaneous lupus lesions (74) (Figure 2). In addition, it has been reported that neutrophils from SLE patients contained elevated levels of oxidized mitochondrial DNA compared with healthy controls (75). Those neutrophils could not degrade the DNA by mitophagy or lysosomal degradation but eventually extruded the oxidized mitochondrial DNA upon stimulation with RNP autoantibodies (76). The modified mitochondrial DNA was a potent immune stimulus for plasmacytoid dendritic cells (76) and was suggested to induce a STING dependent systemic type I IFN induction in mice (75).

Furthermore, accumulation of intracellular cytoplasmic DNA has been observed in epithelial cells of cutaneous lupus lesions (77) (Figure 2). Normally, this DNA should be eliminated by TREX1. However, UV-induced oxidative DNA-modification has been shown to render DNA resistant to degradation by the cytoplasmic DNAse TREX1 (78). UV-irradiation is one of the most important trigger factors for the discoid and tumidus forms of cutaneous lupus and relevant for induction of acute and subacute cutaneous lupus and SLE. Impaired restriction of modified DNA in the cytoplasm upon UV-irradiation could therefore be a relevant disease trigger factor and pathogenic mechanism for cutaneous lupus. Deficiency of TREX1 due to heterogeneous mutations is an additional risk factor for the development of photosensitive forms of SLE (36). In those patients, DNA accumulates in the cytoplasm due to incomplete restriction by TREX1 and can trigger the cGAS-STING pathway leading to type I IFN activation (Figure 2).

UV irradiation is also relevant in the pathogenesis of lupus in patients with SLE and mutations in any of the three subunits of RNase H2 (39). This nuclear enzyme is responsible for eliminating misincorporated ribonucleotides from DNA (79). Mutations that impair this function lead to an enhanced number of ribonucleotides in DNA. Ribonucleotide containing dsDNA has different steric properties that facilitate the formation of cyclobutanpyrimidine dimers in DNA upon UV-irradiation (39). These cyclobutanpyrimidine dimers are among the most frequent UV- induced DNA lesions that need to be eliminated by the DNA repair machinery (80). In RNaseH2 deficient cells from lupus patients UV irradiation causes enhanced DNA damage that consequently led to a prolonged and elevated type I IFN response especially if other trigger factors such as extracellular nucleic acids are present (39) (Figure 2).

Activation of the type I IFN pathway is a hallmark of all cutaneous lupus lesions and can be demonstrated by activation of myxovirus resistence protein A (MXA) and the expression of type I IFN induced chemokines such as CXCL10 in the skin. Keratinocytes from SLE patients have been shown to be primed by type I IFNs suggesting continuous type I IFN production in the skin of lupus patients (81, 82). Type I IFNs can also induce expression of inflammasome components such as AIM2 and IFI16. Inflammasome activation in keratinocytes *in vitro* can lead to upregulation IL-18. This cytokine is detectable in lupus lesions and can induce MHC class II expression and CXCL10 upregulation (83). However, inflammasomes also negatively regulate the type I IFN pathway (84). The relevance of these interactions for cutaneous inflammation in lupus is not fully understood.

Chemokines like CXCL10 have the potential to recruit T cells into the skin (4, 85) (Figure 2). Their influx is accompanied by histiocytes, dendritic cells and eventually neutrophils, which fuel the immunologic response and lead to tissue destruction, swelling, and erythema.

In conclusion, stimulation of an immune response based on activation of innate nucleic acid sensors in tissue resident or immune cells of the skin seems to be an important pathogenic pathway in the induction of cutaneous lupus and might have implications for disseminating systemic disease. The understanding of pathogenic concepts in lupus might allow a categorization of disease subtypes based on etiology. Phenotype genotype correlations revealed that patients with genetic defects in complement components or DNAse I leading to an enhanced prevalence of extracellular DNA are often affected by severe discoid lupus, exanthemas, and prominent kidney involvement (20). Patients with mutations in TREX1 accumulate intracellular DNA and frequently suffer from chilblain lupus but less frequent organ involvement (50, 86). Future detailed genetic analysis in random lupus patients might substantiate this subtyping and help to develop individual therapies. The currently used drugs include corticosteroids, hydroxychloroquine, and methotrexate (3, 87). They have broad anti-inflammatory effects but were not approved for cutaneous lupus. Hydroxychloroquine impairs nucleic acid sensing by building complexes with DNA or RNA (88). It had effectivity in a population of patients with cutaneous lupus and type I IFN signature whereas hydroxychloroquine refractory patients harbored increased numbers of TNF alpha secreting myeloid dendritic cells and required additional quinacrine treatment (89). This further indicates the clinical variability of the immunologic response in cutaneous lupus patients and underlines the need for detailed pathogenic exploration and personalized medicine. Inhibitors of type I IFN and type I IFN receptor have shown clinical efficacy for cutaneous lupus in phase II clinical trials (90, 91). Furthermore, the janus kinase inhibitors that interfere with signal transduction of the type I IFN receptor have shown effectivity in single patients with cutaneous chilblain lupus (47, 52, 92). These therapeutic efforts may pave the way for new treatment options in future. A detailed understanding of disease pathogenesis is a prerequisite for this development.

## Author Contributions

CG wrote the text and designed the figures.

### Conflict of Interest Statement

The author declares that the research was conducted in the absence of any commercial or financial relationships that could be construed as a potential conflict of interest.
